# Unraveling individual differences in learning potential: A dynamic framework for the case of reading development

**DOI:** 10.1016/j.dcn.2024.101362

**Published:** 2024-03-02

**Authors:** Milene Bonte, Silvia Brem

**Affiliations:** a Department of Cognitive Neuroscience and Maastricht Brain Imaging Center, Faculty of Psychology and Neuroscience, Maastricht University, Maastricht, the Netherlands; b Department of Child and Adolescent Psychiatry and Psychotherapy, University Hospital of Psychiatry Zurich, University of Zurich, Switzerland; c Neuroscience Center Zurich, University of Zurich and ETH Zurich, Switzerland; d URPP Adaptive Brain Circuits in Development and Learning (AdaBD), University of Zurich, Zurich, Switzerland

**Keywords:** Dynamic Assessment, Individual differences, Longitudinal brain imaging, Learning, Reading acquisition, Developmental dyslexia

## Abstract

Children show an enormous capacity to learn during development, but with large individual differences in the time course and trajectory of learning and the achieved skill level. Recent progress in developmental sciences has shown the contribution of a multitude of factors including genetic variation, brain plasticity, socio-cultural context and learning experiences to individual development. These factors interact in a complex manner, producing children's idiosyncratic and heterogeneous learning paths. Despite an increasing recognition of these intricate dynamics, current research on the development of culturally acquired skills such as reading still has a typical focus on snapshots of children’s performance at discrete points in time. Here we argue that this ‘static’ approach is often insufficient and limits advancements in the prediction and mechanistic understanding of individual differences in learning capacity. We present a dynamic framework which highlights the importance of capturing short-term trajectories during learning across multiple stages and processes as a proxy for long-term development on the example of reading. This framework will help explain relevant variability in children’s learning paths and outcomes and fosters new perspectives and approaches to study how children develop and learn.

## Introduction

Adaptive and goal-directed learning processes are driving forces of development. Paradoxically, learning-related behavioral and neural changes during skill acquisition in children remain largely understudied. Historically, this can be placed within a longstanding tradition in developmental psychology to view learning as a by-product, rather than an explanatory factor for development (Piaget, 1964). From a practical point of view, learning experiments in children come with multiple challenges, including the design of age-appropriate learning tasks, tailored techniques for the acquisition of functional and anatomical brain data, the need for dense (longitudinal) data sampling across the time course of learning, and efforts to keep children attentive and motivated (Klapwijk et al., 2021, Telzer et al., 2018, van Atteveldt et al., 2021). The actual modeling of learning-related behavioral and neural changes further requires sophisticated analysis techniques that can deal with multifactorial, often incomplete, relatively noisy and variable data. As a result, the vast majority of developmental studies have investigated children’s momentary skills and knowledge at specific developmental stages of interest. Such static performance measures, as commonly assessed in various domains, however quantify the product of learning rather than the learning process or learning potential per se. As a result, these measures are substantially influenced by contextual factors, test-taking skills and test taking situations (Fuchs et al., 2008, Grigorenko and Sternberg, 1998). Moreover, the use of standard static measures makes it challenging to effectively differentiate meaningful inter-individual variability in development from arbitrary, momentary variability. Considering the intricate and interactive nature of behavioral and brain processes that shape individual learning trajectories, a comprehensive and dynamic multi-level characterization of individual children’s short-term changes in learning over time is essential to advance our understanding on both typical and atypical variability, as well as predicting long-term outcomes (Siegler, 2005, Vygotsky and Cole, 1978). Here, short-term refers to changes during a given learning task, where the exact duration will differ depending on the specific skill, paradigm, and/or age of the child. In our dynamic framework, we argue for (re)shifting our attention towards in-depth analyses of these short-term learning processes and trajectories. This aligns with the “microgenetic” or rather microdevelopmental approach as initially proposed by Inhhelder (Inhelder et al., 1992), which asserted that the best way to determine how a child learns is to follow them closely *while they are learning* (Siegler, 2005). Crucially, we suggest to explicitly link these short-term learning processes with long-term developmental changes across multiple levels, employing diverse approaches that track not only (cognitive) behavioral but also functional and structural brain changes. In this context, '*dynamic*' encompasses both the *alterations that unfold within a short timeframe during learning tasks* as well as those *during the long-term evolution of learning with development.* Continuous or intermittent data collection is thus necessary to effectively monitor the dynamic shifts that occur in brain activity and behavior as individuals engage in learning activities, complemented with longitudinal data sampling across development to capture the full scope of the learning process.

We focus on the domain of reading, and discuss some of the main factors that contribute to the formation and continuous change of the neural networks underlying individual differences in cognitive performance. We elaborate on how studies of momentary skills have been pivotal in characterizing different stages of cortical specialization that parallel reading acquisition in groups of typical and atypical readers, including children with developmental dyslexia (Brem et al., 2010, Chyl et al., 2019, Chyl et al., 2021a, Dehaene-Lambertz et al., 2018, Di Pietro et al., 2023, Romanovska et al., 2022). So far however, these studies did not capture children’s highly heterogeneous learning rates and achieved reading levels, or the underlying neuro-cognitive mechanisms . We argue that it is time to develop learning paradigms that enable understanding and predicting individual children’s potential for learning to read beyond their current knowledge and skills. We thus propose a novel and dynamic learning perspective in reading and dyslexia research through paradigms that enable zooming into short-term behavioral and neural changes while children acquire foundational reading skills.

## The developing reading network

Most developmental cognitive neuroscience research involves cross-sectional or longitudinal measurements of long-term developmental changes, i.e., changes across multiple months or years. These studies show that children’s idiosyncratic development is shaped by interactive influences across various levels of change, ranging from genetic to brain structure and function, behavior, cognition, and (socio-cultural) environment (Bronfenbrenner, 2005, Miller, 2009, Sameroff, 2010). Recent advances in developmental sciences have further highlighted the pivotal role of various interacting emerging functions such as language, reading, math, socio-emotional skills, and executive function, in shaping children's diverse learning outcomes (Johnson, 2011, Kievit, 2020, van Atteveldt et al., 2021). All these factors act on the brain in the course of ontogeny and shape the developing cognitive brain networks (Johnson et al., 2015).

As a culturally acquired skill, learning to read is a complex and lengthy process that typically begins with formal enrollment in school. By that time, a child has already acquired the basic linguistic and cognitive skills necessary to learn to process written information. These early learning experiences, including established reading precursor skills (e.g., phonological awareness, letter knowledge, word retrieval or vocabulary), and domain general executive functions such as attentional and working memory skills, show high inter-individual variability and depend on various biological, and contextual factors. Consequently, early learning experiences critically influence the rate of initial reading acquisition (Caravolas et al., 2012, O’Brien and Yeatman, 2021, Thompson et al., 2015), together with various aspects of a written language's complexity, such as the writing system (e.g., alphabetic, syllabic, morphosyllabic) or the level of orthographic depth such as for example the opaque English vs. transparent Finnish orthography (Landerl et al., 1997, Seymour et al., 2003, Ziegler and Goswami, 2005).

What is common to reading acquisition in all languages is the reliance on an extended and complex brain network that with experience and maturation successively allows for efficient processing of written material (Brem et al., 2010, Chyl et al., 2019, Dehaene-Lambertz et al., 2018, Di Pietro et al., 2023, Linkersdörfer et al., 2015, Wang et al., 2017, Yeatman et al., 2012a). Unlike the network responsible for processing spoken language, which has co-evolved with humankind, reading is a recent skill in evolutionary terms and therefore cannot rely on brain structures specifically evolved for text processing. Instead reading relies on the establishment of culturally defined connections between spoken and written language. The formation of these connections requires plastic brain changes and adaptations that occur during development, learning and extensive practice (Dehaene and Cohen, 2011). The resulting reading network of - mainly left hemispheric - brain regions, essentially comprises an indirect dorsal stream and a direct ventral stream for processing print (Jobard et al., 2003, Kearns et al., 2019, Pugh et al., 2001). The function of this network is further modulated by attentional circuits with which it is tightly connected (Chen et al., 2019, Vogel et al., 2012). Brain regions of the dorsal stream include the left temporo-parietal and the inferior frontal cortex, connected through the arcuate fasciculus. The temporo-parietal lobe is relevant for decoding, phonological processing, auditory word form processing and recognizing word meanings (Jobard et al., 2003, Romanovska and Bonte, 2021). The frontal lobe contributes to information sequencing, motor planning and higher-level cognitive functions such as attention, working memory, and executive control (Houdé et al., 2010, Richlan et al., 2009). This dorsal stream develops early during reading acquisition. It enables initial reading through word decoding by linking letters to speech sounds (Jobard et al., 2003, Romanovska and Bonte, 2021). Evidence from short-term learning paradigms, such as symbol - speech sound association learning and text-based recalibration, specifically implicate the temporo-parietal cortex during the early phases of establishing letter-speech sound associations (Bonte et al., 2017, Wise Younger and Booth, 2018). The ventral stream comprises the ventral occipito-temporal cortex critical to process perceptual and lexical aspects of visual word forms (Caffarra et al., 2021, Cohen et al., 2000, Lerma-Usabiaga et al., 2018, Vinckier et al., 2007) and which is structurally connected to temporal and inferior frontal regions via the inferior longitudinal fasciculus and the inferior frontal-occipital fasciculus (Kearns et al., 2019). Through connections to temporal language and fronto-parietal areas the ventral occipito-temporal visual word form system may act as a gateway to link attention and language functions, critical for reading (Chen et al., 2019). While theoretical accounts of reading assume parallel involvement of the dorsal and ventral streams in proficient readers (Coltheart et al., 2001), the ventral stream is considered particularly relevant for fluent reading of familiar/frequent words through the processing of morphological units and direct lexical access (Pugh et al., 2001, Yablonski et al., 2019). Accordingly, a developmental shift toward reliance on the ventral stream has found support in neuroimaging studies of connectivity and activity in the reading network (Church, 2008, Di Pietro et al., 2023, Wise Younger et al., 2017).

The brain’s reading network and its two processing streams demonstrate considerable convergence across different languages and writing systems (Bolger et al., 2005, Rueckl et al., 2015) and this remarkable cross-cultural consistency is evident from early stages of reading skill acquisition (Chyl et al., 2021a, Chyl et al., 2021b, Feng et al., 2020) and becomes more pronounced with increasing expertise (Zhang et al., 2023). Nevertheless, minor variations across languages and writing systems have been reported (Paulesu et al., 2000, Tan et al., 2001). The orthographic transparency of languages and the characteristics of the writing system could influence the relative engagement of the dorsal and ventral streams. Alphabetic languages with transparent letter-to-sound correspondences (e.g., Finnish and Italian), tend to rely more on phonological decoding and, consequently, on the dorsal route in contrast to more opaque (e.g., English) orthographies (Oliver et al., 2017). Morpho-syllabic writing systems, such as Chinese, seem to rely more on lexical access from orthographic input, potentially favoring the ventral stream (Bolger et al., 2005, Tan et al., 2005).

Within the reading network, we thus see a different weighting of its subcomponents depending on the individual’s age and skill level, the diverse monolingual or multilingual language experiences, language characteristics (e.g., orthographic transparency) and reading strategies. In the increasingly common case of multilingualism, the combination of languages, their typological distances, the level of proficiency, and age of acquisition, among other factors, significantly impact network characteristics and influence language development (Jasinska and Petitto, 2013, Kepinska et al., 2023, Mechelli et al., 2004). In essence, this variance in the weighting of dorsal vs. ventral reading streams, along with the structural and functional characteristics of the broader language networks, significantly shapes children's diverse neurobehavioral outcomes and learning trajectories.

Maturation and reading-experience dependent alterations have likewise been shown in patterns of white-matter connectivity across anterior and posterior cortical areas of the reading network (Huber et al., 2018, Vanderauwera et al., 2018, Yeatman et al., 2012b). Longitudinal data show a reciprocal relationship between white matter alterations and reading proficiency (Roy et al., 2022). Although much less studied, also subcortical areas such as the thalamus, striatum or the cerebellum, contribute to reading, reading acquisition and variations in reading skills. While still being debated, the cerebellum may play a predictive role in language processing, such as during sentence reading (Diedrichsen et al., 2019, Moberget et al., 2014). It is also assumed to exert a modulatory influence on both the dorsal and ventral streams, thereby contributing to variations in sub-lexical phonological and lexical processing (Alvarez and Fiez, 2018) and thus also potentially to the development of dyslexia (Nicolson et al., 2001). The thalamus plays a pivotal role as an adaptive sensory gateway and filter, responsible for relaying sensory information to higher order processing areas, including the prefrontal cortex. It selectively fine-tunes attention towards pertinent sensory information, effectively regulating the focus of attention. This gating mechanism is likely achieved by functional inhibition of activity, manifested as alpha band oscillations (8–13 Hz) and subsequent routing of information flow to task relevant regions. This results in heightened gamma band synchronization (30–100 Hz) and alpha band desynchronization in these regions as observed in EEG/MEG recordings (Jensen and Mazaheri, 2010). With regard to natural reading, alpha oscillations have been suggested to function as an attentional gating mechanism, coordinating the interaction between oculomotor (saccades) and visual systems and thereby shaping the flow of visual information (Pan et al., 2023). Deficits in attentional processes or alterations in the underlying brain network characteristics, such as in the cortico-thalamic auditory or visual pathways (Müller-Axt et al., 2017, Tschentscher et al., 2019, Žarić et al., 2018) may thus explain variability in e.g., letter-speech sound learning success and reading impairments (Díaz et al., 2012, Guerra et al., 2023) and the frequent comorbidity of dyslexia and attention-deficit hyperactivity disorder (Jagger-Rickels et al., 2018). Striatal contributions to reading are particularly pertinent during the initial phase of reading acquisition, as implicit statistical processes are utilized to learn the associations between graphemes and phonemes (Hancock et al., 2017, Elleman et al., 2019, Siegelman et al., 2020). Their contributions may also remain relevant later on, when compensatory mechanisms emerge in impaired readers (Hancock et al., 2017).

The multiplex contributions of cortical and subcortical areas also explains that any variation or alteration in its constituent parts with regard to e.g., size, activation or connectivity can affect the developing reading network. For example, micro-damage to the ventral occipito-temporal cortex or altered activation, structural or functional connectivity between areas go along with difficulty in fluent reading (Pleisch et al., 2019a; Di Pietro et al., 2023, Pflugshaupt et al., 2009, Richlan et al., 2009, van der Mark et al., 2011, Yeatman et al., 2012a, Wang et al., 2020a, Wang et al., 2020b). Alternatively, specific brain network characteristics may also contribute to resilience, for example, by supporting compensatory mechanisms (Zuk et al., 2021). This diversity in alterations, deficiencies, and weighting of different subcomponents of the reading network, as previously discussed, supports a multifactorial framework with dynamic, reciprocal interactions that accounts better for the individual variability in learning observed in developmental dyslexia than a singular, unified deficit explanation (Menghini et al., 2010, O’Brien and Yeatman, 2021, Peterson and Pennington, 2012).

## Multiple interactive sources of individual differences

**Fig. 1:**
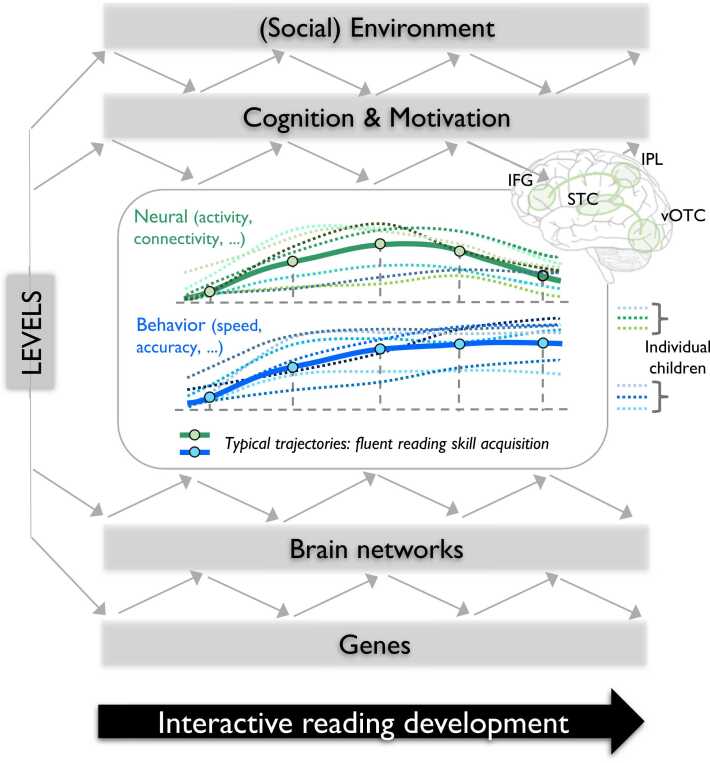
Schematic illustration of children’s individually variable neural and behavioral reading trajectories. Multiple interactive levels (genes, brain networks, cognition, motivation, and social environment) dynamically shape children’s idiosyncratic trajectories of reading development. Hypothesized typical neural and behavioral reading trajectories (bold lines) are illustrated together with examples of individual children’s trajectories (dotted lines). Neural trajectories refer to functional (activity, connectivity) and anatomical (area, connectivity) developmental changes; Behavioral trajectories refer to changes in speed and/or accuracy of reading skills. The developmental timing of these trajectories will differ for different reading skills (e.g., letter-speech sound mapping, visual letter, word recognition). Main regions of the brain’s reading network: STC = superior temporal cortex, vOTC = ventral occipito-temporal cortex, IPL = inferior parietal cortex, IFG = inferior frontal cortex.

Children’s idiosyncratic trajectories of reading development are shaped by reciprocal interactions across multiple levels including genes, brain networks, cognition, motivation, and social environment (Fig. 1)*.* The interplay between genes, their interactions, epigenetic modifications, and environmental factors significantly contributes to the development of neural systems that enable mental functions and learning throughout life (Bueno, 2019, Gialluisi et al., 2019). While various mental functions show a moderate to high heritability (Bueno, 2019), the individual impact of single genes on explaining variation is generally small. Despite the identification of numerous dyslexia candidate gene loci, the current understanding of polygenic risk scores only explains around 6% of the variability in reading ability (Doust et al., 2022). Recent genome-wide association studies on the other hand have revealed that specific traits like word reading, nonword reading, nonword repetition, spelling, and phonological awareness have a heritability ranging from 10% to 25% attributed to single-nucleotide polymorphisms (Eising et al., 2022, Georgitsi et al., 2021). One potential mechanism of how specific genes may contribute to the etiology of developmental dyslexia and reading skills is through alterations in neuronal migration during the early stages of cortex formation in brain development, as demonstrated in animal studies (Centanni et al., 2014, Galaburda et al., 1985, Meng et al., 2005, Paracchini et al., 2006; see Guidi et al., 2018 for critical considerations). Such altered neuronal migration could explain the observation of cortical micro-abnormalities (ectopias and displasias) within the language network in individuals with dyslexia (Galaburda et al., 1985, Galaburda et al., 2006). Furthermore, specific microstructural alterations, including changes in gray matter volume, surface area, and white matter structure within the neural networks for language processing and reading, have been associated with particular genes (Carrión-Castillo et al., 2023, Darki et al., 2012, Skeide et al., 2016). Genes associated with neuronal migration deficits may also influence the cortical excitability by impairing the formation of neural excitatory-inhibitory circuits. This disruption can increase neural noise and disturb the temporal precision and synchronicity of circuit function during auditory and visual processing, potentially contributing to the deficits observed in reading and reading precursor skills (Hancock et al., 2017).

Environmental factors, some of which are also dependent on socio-economic status (SES) of the family, e.g., home language and literacy environment, and parental educational level, as well as perinatal or health problems, stress or screen-based media-use in early childhood, have all been linked to reading abilities (Hutton et al., 2020, Khanolainen et al., 2020, Theodoridou et al., 2021, van Bergen et al., 2015). A clear separation of genetic and environmental influences on children’s brain network formation and individual learning trajectories is difficult as children and their environments interact, and their individual characteristics and interests, related to their genotype, can induce active, passive, or evocative changes in the environment (Hart et al., 2021). For instance, children's home literacy environment, which includes reading activities, books at home, and library visits, depends on parents' reading attitudes but can be adjusted by the parents or the child based on their specific interests and needs. Therefore, changes in environmental contexts that may occur in different phases of development, either independently (e.g., the onset of formal schooling) or as an interaction with genotype (e.g., niche selection), have a significant impact on individual learning trajectory variability at multiple time points. Changing environmental conditions in turn will affect heritability. For example, heritability of reading performance increases with age, likely due to a decrease in environmental differences with access to education and formal schooling and increased niche selection based on children's individual abilities (Logan et al., 2013, Peterson and Pennington, 2015). Similarly, dyslexia has a stronger genetic basis in children from higher SES and higher-educated parents than in those from lower SES families or in children with higher cognitive abilities (Peterson and Pennington, 2015).

Another key factor influencing children’s short and long-term learning trajectories involves their goal settings, active engagement and expected rewards. Naturalistic learning results from cyclic interactions between perception, action and motivation (e.g., Fuster, 2001, Fuster, 2017; Kraus and White-Schwoch, 2015). The trajectories of children's response accuracy during a 20-minute symbol-speech sound learning task were recently found to be influenced by task instructions. Higher accuracies were observed during the later phases of the task for children who received goal-directed instructions compared to those who received implicit task instructions (Verwimp et al., 2023). Arguably, this enhancement may result in the formation of qualitatively different neural representations of symbol-sound pairs (Pleisch et al., 2019b), similar to attentional engagement and reward related enhancements of visual cortical representations in adults (Schiffer et al., 2014) and non-human primates (Arsenault et al., 2013). Alternatively it has also been argued that altered implicit use of statistical contingencies to form visual-phonological associations, or differences in implicit memory formation and memory decays may explain differences in reading success (Jaffe-Dax et al., 2017, Jones et al., 2018, Wang et al., 2015). In fact, developmental dyslexia may come with a reduced sensitivity to the statistical structure of speech (Bonte et al., 2007, Noordenbos et al., 2013, Vandermosten et al., 2018) and sound in general (Gabay et al., 2015), or alterations in neural adaptation and priming processes (Bonte and Blomert, 2004, Jaffe-Dax et al., 2017; Perrachione et al., 2016; Peter et al., 2019; Pugh et al., 2008; Ozernov-Palchik et al., 2022). But these findings are not always replicated (see Chyl et al., 2023; Elleman et al., 2019).

Existing research on motivation-performance interactions across longer developmental time-scales has primarily concentrated on higher level reading skills including sentence and text comprehension. While these motivation-performance interactions were often shown to be bi-directional (Hebbecker et al., 2019, Schiefele et al., 2016), motivation may follow performance improvement more strongly than vice versa (Miyamoto et al., 2018, van Bergen et al., 2021, van Bergen et al., 2023). Motivation-performance interactions will thus be different for individual children and may change with individual differences in performance gains across development. A better understanding of these short- and long-term interactions and their relation to genetic and environmental factors requires going back to the earliest stages of reading development, and subsequent longitudinal follow-up of reading gains and outcomes in children across diverse backgrounds. This is particularly important because children’s reading skills (Petscher et al., 2017), and their ability to self-regulate their learning (Burnette et al., 2013, Dweck and Leggett, 1988) are profoundly impacted by their mindset, which refers to their beliefs about their potential to improve their abilities through time and effort. Children with dyslexia, learning difficulties or those from disadvantaged SES backgrounds often display lower reading self-beliefs (Baird et al., 2011, Claro et al., 2016, Destin et al., 2019), which can negatively affect their reading development.

## Nonlinear and dynamic neural learning trajectories

**Fig. 2:**
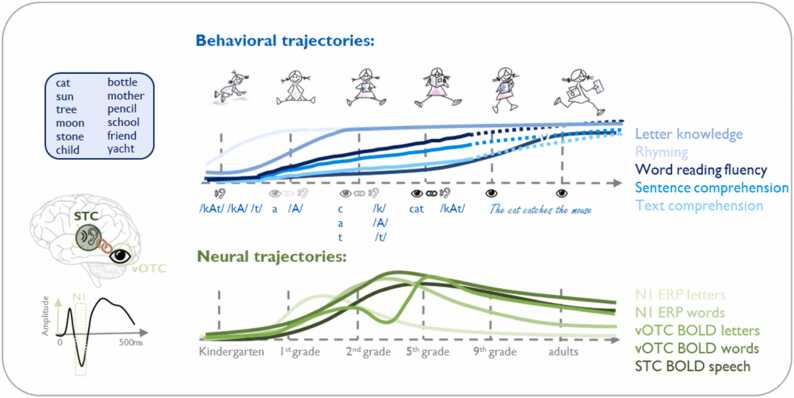
Behavioral and neural learning trajectories in the context of reading acquisition. *Upper part*: schematic representation of the behavioral learning trajectories for different skills such as rhyming, letter-sound knowledge, reading fluency, sentence and text comprehension. As an example, the time-point at which letter-sound knowledge reaches the ceiling depends on the orthographic transparency and is typically achieved within a few months of schooling in orthographically shallow languages like German, Dutch, or Finnish. *Lower part:* schematic representation of neural activation trajectories across development, including trajectories for sensitivity to letter (Di Pietro et al., 2023, Fraga-González et al., 2021) and word or sentence processing (Brem et al., 2009, Dehaene-Lambertz et al., 2018, Feng et al., 2022, Maurer et al., 2006, Maurer et al., 2011, Ozernov-Palchik et al., 2022) reflected by the left occipito-temporal visual event-related potential N1 after around 200 ms (shown in the waveform graph at the bottom left) and vOTC activation. The dark green line represents the trajectory of the BOLD signal in the superior temporal cortex (STC) during audiovisual processing of speech sounds (Di Pietro et al., 2023, Romanovska et al., 2022). Note, not for all illustrated measures, longitudinal data is available for the whole time-course. STC: superior temporal cortex; vOTC: ventral occipito-temporal cortex, BOLD: blood oxygenation dependent signal; ERP: event-related potential.

One of the challenges in characterizing the course of functional specialization during skill learning is the observation that neural changes are often nonlinear, and not necessarily in direct alignment with the observed behavioral gains (Dehaene-Lambertz et al., 2018, Di Pietro et al., 2023, Feng et al., 2022, Fraga-González et al., 2021, Fraga-González et al., 2022, Huber et al., 2018, Karipidis et al., 2021, Romanovska et al., 2022, Pugh et al., 2008). This accounts for long-term changes over months and years of learning and is also presumed to mirror changes in short-term learning as detailed below. Reading acquisition provides an excellent example to study and characterize neural and behavioral trajectories of academic skill learning, both in the short term, by focusing on learning specific components relevant to the reading process, and in the long term, by following children from the preliterate stage in preschool through many months and years to the fluent and expert reading stage. Similar to behavioral learning curves for basic motor and perceptual skills (Kami et al., 1995, Xu et al., 2009, Yotsumoto et al., 2008), developmental growth curves for reading related competences such as letter knowledge (Fraga-González et al., 2021), word reading fluency (Francis et al., 1996) and reading comprehension (Lervåg et al., 2018) are characterized mostly by logarithmic trajectories with an initial exponential increase followed by asymptotic stability with gained expertise (Fig. 2). Brain measures reflecting functional or structural growth curves of specific components of the developing reading network instead often exhibit an inverted U-shape (Di Pietro et al., 2023, Fraga-González et al., 2021, Maurer et al., 2006, Romanovska et al., 2022). Similar behavioral and neural trajectories are also evident during short-term learning tasks of language and reading skills (Guerra et al., 2023, Hashimoto and Sakai, 2004, Malins et al., 2021, Mayringer and Wimmer, 2000; Steacy et al., 2021; Verwimp et al., 2023; Zhang et al., 2021). These short-term trajectories may be affected by individual differences in reading level (Guerra et al., 2023, Malins et al., 2021, Mayringer and Wimmer, 2000, Pugh et al., 2008; Steacy et al., 2020; Zhang et al., 2021) and attention skills (Guerra et al., 2023), as well as by task instructions (Verwimp et al., 2023), and linguistic features, such as the orthographic-phonological regularity (Steacy et al., 2021), and semantic features (Steacy et al., 2020) of the materials being learned. The trajectories of learning and recall are further modulated by sleep consolidation processes, as evidenced by studies that have assessed the effects of naps or overnight sleep on various types of learning, including vocabulary acquisition (Henderson et al., 2021, James et al., 2020, Landi et al., 2018, Smith et al., 2018), pseudoword learning (Malins et al., 2021), auditory sequence learning (Ballan et al., 2023), or mirror-letter differentiation (Torres et al., 2021). A better understanding of the mechanisms underlying diverse learning trajectories thus may provide a more accurate view on the individual learning potential, later outcomes such as reading competence and inform on intervals when individuals may benefit most from support or intervention. Hereby it seems especially important to distinguish relevant from irrelevant (noise) variability in the trajectories of brain and behavioral measures. Such information is crucial to advance early prediction and to reliably distinguish children with typical or poor reading outcomes at an early stage. Here we discuss how established models of perceptual and motor learning can be used to better understand the mechanisms underlying nonlinear behavioral and neural learning trajectories of reading.

From a neurobiological point of view, different models have been proposed to explain non-linear trajectories of perceptual and motor learning. Building on the seminal Hebbian principle of “fire together, wire together” (Hebb, 1949) as one of the bases of learning, research points to additional adaptive mechanisms of feedforward, feedback and moderating signals in cortical neurons (Chéreau et al., 2022, Magee and Grienberger, 2020). Together these mechanisms interactively tune the strength and weighting of synapses and establish efficient local and global circuits enabling improvements in behavioral performance (Chéreau et al., 2022). With motor learning for example, increases in synaptic density during the intense early learning phase, and subsequent stabilization of newly formed synapses, are followed by selective reduction of synaptic density with further learning (Lövdén et al., 2020, Xu et al., 2009). Plastic changes on the synapse level thus correspond to an invertedU-shaped curve with its maximum during the intense early learning phase and leveling off with further training and expertise. Similar trajectories of change were also observed in early studies on cortical volume change during spatial navigation or motor (juggling) learning in humans (Draganski et al., 2004, Maguire et al., 2000). But only recently studies with dense, repeated neuroimaging are starting to show the full trajectory and dynamics of cortical volume changes (Wenger et al., 2017) in humans, with a learning-related initial growth and subsequent decline in cortical volume. The *expansion-exploration-selection-refinement* theory (Lindenberger and Lövdén, 2019, Lövdén et al., 2020, Wenger et al., 2017) converges these insights from microscopic and macroscopic findings on skill learning to explain mechanisms underlying the nonlinear, inverted U-shaped trajectories in biological terms. Accordingly, an initial phase of expansion, resulting in increased synaptic density on the microscopic level or larger cortical volumes and stronger activation and/or functional connectivity on the macroscopic level, allows for exploration of various neurocognitive circuitries to improve performance. This phase is characterized by high behavioral and neural inter-trial variability which then declines during selection and stabilization of the optimal neural circuitry for a given function through reinforcement learning. With further practice, these circuitries are refined by removal of unselected synapses and accompanied by a reduction of cortical volume, activation and a decrease in intertrial variability.

Changes in biological measures as predicted by the *expansion-exploration-selection-refinement* model largely correspond to learning trajectories predicted by computational models of predictive coding (Friston, 2010). These models provide an explanation of how the integration and matching of feedforward and feedback information in specific brain regions result in expectations, predictions and prediction errors that guide learning and are encoded as neural firing rates and patterns of brain activity on micro- (Schultz and Dickinson, 2000) or macroscopic levels (Price and Devlin, 2011, Zhao et al., 2019). Several studies suggest that these mechanisms reported for motor-perceptual processing also apply to higher cognitive functions such as reading (Fig. 2). For example, the development of sensitivity to print (letters or words) as measured with visual event-related potentials of the EEG and/or corresponding fMRI activity of the visual word from system in the left occipito-temporal cortex (Dehaene-Lambertz et al., 2018, Fraga-González et al., 2021, Maurer et al., 2006) showed inverted U-shaped changes with children’s reading gains over months or years of practice. Likewise the activity in the posterior superior temporal cortex during audiovisual print-speech sound integration showed similar developmental trajectories (Di Pietro et al., 2023, Romanovska et al., 2022). In the context of letter learning, the peak activity in the visual ERP, observed longitudinally from pre-reading stages in kindergarten to advanced reading stages in the fifth grade, was noted approximately six months into schooling (Fraga-González et al., 2021, Fraga-González et al., 2022). This peak coincides with the phase when children intensively practice and establish letter-speech sound associations. Concurrently, speech sound processing was found to significantly increase activity in the superior temporal cortex, indicating a refinement of phoneme representations during reading acquisition (Di Pietro, Karipidis, et al., 2023). When processing more complex visual stimuli, such as whole words, sentences or intricate audiovisual associations, a similar but slightly delayed inverted U-shaped developmental trajectory of activity is anticipated (Maurer et al., 2006, Ozernov-Palchik et al., 2022, Romanovska et al., 2022). This concept is also illustrated in Fig. 2. However, it is important to note that, unlike for EEG measures, there is still a lack of longitudinal fMRI studies that track word and/or sentence processing from pre-reading to advanced reading stages (Chyl et al., 2021a, Chyl et al., 2021b). The assumptions thus rely on cross-sectional investigations and longitudinal studies covering only a specific interval during learning (Brem et al., 2009, Dehaene-Lambertz et al., 2018, Feng et al., 2022, Maurer et al., 2011, Ozernov-Palchik et al., 2022). Given the presumed parallel between short- and long-term learning trajectories, we expect similar variability in short-term trajectories during learning tasks of component skills of reading (within minutes or hours). Data from both EEG and fMRI studies examining learning tasks of letter-speech sound associations or speech structure indeed show that the change in activity of the brain through learning occurs rapidly and nonlinearly (Brem et al., 2018, Karipidis et al., 2018, Pleisch et al., 2019b, Zhang et al., 2021). Moreover behavioral and/or neural variability during short-term learning procedures, such as the peak of the learning curve or slowing of learning speech structures or audiovisual associations, was found to account for additional variability in current or future reading skills as compared to total outcome scores (Guerra et al., 2023, Karipidis et al., 2018, Zhang et al., 2021).

In summary, there is evidence, such as for the development of visual and audiovisual letter and speech sound processing, that the maxima of the non-linear activation changes coincide with a behavioral phase of intense learning, which may involve biological expansion and high prediction error signaling. More advanced approaches to characterize short-term learning in behavior and brain such as through the use of computational modeling to derive trial-wise parameters of learning (Fraga-González et al., 2023) or more sophisticated statistical analyses incorporating the shape of learning curves (Curran et al., 2010) could significantly deepen our understanding of the variability in skill acquisition and their relevance for long-term learning as suggested within our dynamic framework.

## Early prediction and the need for dynamic measures

While the importance of early prediction of academic outcomes, and of reading problems in particular, has been recognized for at least 50 years (Jansky and De Hirsch, 1972), we still lack accurate prospective measures. Here ‘early’ refers to early enough to prevent an accumulation of reading problems and associated socio-emotional, motivational issues, and to enable timely intervention. Optimally, this is before the start of (word) reading instruction (Ozernov-Palchik and Gaab, 2016, Poulsen, 2018, Wanzek et al., 2013). We are thus faced with the challenge to obtain a reliable estimate of a child’s future performance on a complex cognitive skill that has not been acquired yet and furthermore shows large variability both between individuals and across development. Precisely quantifying children's non-linear learning trajectories during short learning tasks relevant to reading has thus the potential to significantly enhance our ability to predict their long-term reading outcomes.

One important indicator of later reading difficulties is familial risk of dyslexia. Children with a first-degree relative (i.e., parent or sibling) with a clinical diagnosis of dyslexia have 40–50% chance of developing dyslexia (Grigorenko, 2004, Snowling and Melby-Lervåg, 2016, van Bergen et al., 2012), compared to a prevalence of 5–10% in the general population (Blomert, 2005, Shaywitz and Shaywitz, 2008). In pre-readers, familial risk of dyslexia is associated with a neurobehavioral (auditory) phonological deficit (Hakvoort et al., 2015, Snowling and Melby-Lervåg, 2016, Yu et al., 2020;Vandermosten et al., 2020) as well as with poor letter knowledge, poor rapid automatized naming (RAN) and delayed language development (Lohvansuu et al., 2018, Snowling and Melby-Lervåg, 2016). These difficulties, however, can be also present in pre-readers with a familial risk who will not develop dyslexia. Consequently, these early measures do not provide reliable indicators of later reading problems, as they will also identify children who will not develop reading problems.

Similarly, behavioral screening of broader population samples using standardly employed static measurements of children’s reading precursor (also ‘pre-reading’) skills, leads to successful identification of many children who are at risk (true positives) but again also to substantial over-identification (high false-positive rate, Catts and Petscher, 2018; Poulsen, 2018). Bi- or multilingual children and those speaking a minority language for example seem particularly susceptible for misidentification due to the lack of appropriate, standardized language-neutral assessments (Samson and Lesaux, 2009). While precision is improved with multi-factorial assessment of risk/protective profiles across a range of precursors e.g., letter knowledge, RAN, phonological tasks (Catts and Petscher, 2018, Ozernov-Palchik and Gaab, 2016, Verwimp et al., 2020), these profiles still show large heterogeneity (Verwimp et al., 2020). Furthermore, static tests of current skills show high sensitivity to context variables, such as a child’s SES background, motivation, and prior knowledge (Jeltova et al., 2007), and floor-effects due to complex task demands (Catts et al., 2009), which reduces their prognostic value.

To increase precision, we need to develop measures for early prediction that estimate a child’s potential for learning beyond their current knowledge and skills. In fact, in the educational context, research substantiates the application of dynamic assessment (DA) for more accurate prediction and guidance of individual support for children in classroom settings (Cho et al., 2020, Dixon et al., 2023, Gellert and Elbro, 2018). The DA approach measures children’s potential to learn new skills given assistance, i.e., their learning potential or what children can potentially achieve, with the right support. A child's learning potential for a specific reading skill is a dynamic reflection of the intricate factors influencing reading development (including also teaching, intervention, motivation). Hence, conducting dynamic assessments multiple times across development and for specific skills is essential for identifying and addressing evolving learning needs. DA can be reliably performed before the onset of formal (reading) instruction and provides valuable information on a child's ability to learn a specific set of skills, particularly those pertinent to the process of reading acquisition later on. It thereby offers insights into potential challenges a child may encounter while learning and gauges the ease with which a child can acquire these essential skills. Recent experimenter-guided DA of decoding skills (including letter-sound association learning) indeed suggests that children’s future reading levels can be estimated from their obtained summed accuracy scores on learning tasks (kindergartners, Gellert and Elbro, 2017, Gellert and Elbro, 2018), or the number of required instructional prompts (first-graders, Cho et al., 2020). These DA measures have additional predictive power over static tests for children’s reading fluency 2–3 years later both in opaque and transparent orthographies, especially when used during early stages of reading development and for children with diverse linguistic backgrounds (Dixon et al., 2023). Yet, also these dynamic assessments rely on snapshots of learning outcomes rather than the learning trajectories themselves. Consequently, current DA measures of early reading skills are not precise enough to trace rapid changes that occur with learning, and to overcome the practical problems of (over)identification (Poulsen, 2018). Enhanced predictive precision requires valid empirical models of (a)typical variability and dynamic learning measures (e.g., slope and rate of improvement) in addition to total outcome scores.

Ultimately, empirical models of (a)typical variability need to incorporate detailed knowledge of the developing reading network including the multiple genetic, environmental, and motivational factors that shape children’s learning and development. Ideally, these models also account for children’s early learning experiences, and eventually other factors such as early life events, physical development and sleep habits, and are grounded in both behavioral and brain evidence. For example, longitudinal studies suggest that infants’ brain responses to speech sounds are associated with the proficiency of reading skills they later develop (Guttorm et al., 2010, Molfese, 2000, van Zuijen et al., 2012). Furthermore, as compared to behavioral measures only, brain measures have been shown to improve the prediction of individual differences in reading skills in 5–7-year-old pre-readers with/without familial risk of dyslexia (Karipidis et al., 2018, Maurer et al., 2009, Wang et al., 2020a, Wang et al., 2020b) and in older children with/without dyslexia (Hoeft et al., 2007, Hoeft et al., 2011). While brain measures themselves are unlikely to become part of routine screening for reading problems, these findings show that there are objectively measurable characteristics that are not yet captured by current behavioral screening procedures. Precise knowledge of these neural characteristics, will be essential in identifying and understanding relevant variability in behavioral trajectories and outcomes and may, with future technological advancements, contribute to more precise phenotyping and tailoring of interventions to individual needs.

## Methodological approaches and challenges

To investigate children’s learning trajectories, we need tailored experimental paradigms, data acquisition approaches, and statistical analysis techniques that enable modeling both group-averaged changes over time and individual differences in these changes. These methodological approaches benefit from the recent increase in the number of longitudinal studies in developmental cognitive neuroscience (Telzer et al., 2018), including those that follow (Ben-Shachar et al., 2011, Brem et al., 2010, Dehaene-Lambertz et al., 2018, Di Pietro et al., 2023, Ozernov-Palchik et al., 2023) and/or predict (Bach et al., 2013, Karipidis et al., 2018, Maurer et al., 2009, Wang et al., 2020a, Wang et al., 2020b) neuro-behavioral changes with reading development. This shift towards longitudinal studies is particularly relevant in the study of reading because cross-sectional age-group comparisons are confounded by children’s highly variable learning rates, due to, for example, age of school entry, IQ, SES, native language and gender, i.e., boys acquire reading more slowly than girls (Goswami, 2003). In dyslexia, cross-sectional designs are even more problematic because of the absence of appropriate control groups. That is, age-matched controls necessarily differ in reading level and other factors such as verbal IQ, whereas controls matched on reading-level differ in age (and cognitive/brain maturation) and their similar behavioral scores may stem from different cognitive strategies (Goswami, 2003, Karmiloff-Smith, 1998). Longitudinal measurements are crucial to overcome these problems, and ideally involve dense sampling across critical periods of change.

While group-level approaches have been essential in characterizing long-term longitudinal changes in the reading network, due their focus on central tendencies among groups, individual differences within groups are treated as noise. This eases the interpretation of typically highly variable children data because random or task-irrelevant variability, e.g., related to measurement noise, different levels of stress or motivation, is automatically filtered out. At the same time however, meaningful individual variability, related to e.g., person-specific developmental factors, task strategies or compensatory processes, are also eliminated. To understand this variability, group-averaged analyses can be complemented with analyses of inter-individual consistency, via penetrance maps quantifying the number of participants with significant fMRI activation within a given voxel or brain region (Olulade et al., 2020, Seghier and Price, 2016). Moreover, individual variability in the location and possibly the timing of functional specificity needs to be accounted for in region of interest (ROI) analyses, as the use of group-averaged ROI definitions may not adequately capture the unique functional characteristics present in each individual's brain as for example seen in the location of the visual word form area (Feng et al., 2022, Frost and Goebel, 2012, Glezer and Riesenhuber, 2013, Nordt et al., 2021). Inter-subject correlation of brain activity and representational similarity analysis enables studying individual differences in more detail (Jangraw et al., 2023, Nguyen et al., 2019, Thiede et al., 2020).

Reliable modeling of functionally relevant inter-individual variability in children’s learning trajectories is essential because there is no uni-dimensional boundary between typical and atypical variability in reading development (Pennington, 2006, van Bergen et al., 2014). We thus need models that permit distinguishing latent groups that are not directly observed but that must be estimated from the (multidimensional) characteristics of the data while minimizing the risk of producing spurious results. Promising approaches based on machine learning model variability in a given brain-behavior association across a large and phenotypically diverse group of individuals and then quantify the placement of an individual’s data along a normative range (Marquand et al., 2019, Siugzdaite et al., 2020). These approaches then enable modeling an individual’s data both as an outlier along a continuum (non-categorical) and as a qualitative deviation from the normative range (categorical). Other approaches for normative modeling, identify patterns of longitudinal change among latent groups using growth mixture modeling (Ram and Grimm, 2009, Yeung, 2018).

In studying how multiple genetic-to-environmental factors interactively shape longitudinal change, determining cause and effect (or leading and lagging relationships) still poses a particular challenge. Bi-directional influences between factors of interest, such as academic performance and effort, across multiple longitudinal measurements, can be quantified using e.g., cross-lagged panel models (Liu et al., 2023, Usami et al., 2019). Comprehensive theoretical and analytical frameworks for understanding complex multifactorial interactions are provided by dynamic system approaches. In line with our plea to closely examine children’s individual neuro-behavioral learning processes during reading development, these approaches have emphasized the importance of e.g., relating individual children’s cognitive growth patterns to growth patterns in brain development (Fischer, 2008), and nonlinear forms of change emerging from dynamic interactions between cognitive and environmental components of spoken language development (Freeman and Cameron, 2008, van Dijk and van Geert, 2023). Network models provide another type of multifactorial dynamic approach that is increasingly adopted in clinical and developmental psychology (Borsboom et al., 2021). These models excel in estimating and illustrating conditional associations in data without relying heavily on predefined assumptions about the generating model. This makes network models highly adept for exploratory data analysis and visualizing dependency patterns in children’s multifactorial developmental data.

The detailed temporal dynamics of short- and long-term longitudinal learning processes can be quantified through growth models, which are particularly powerful for tracking and modeling specific variables over time (Curran et al., 2010). Growth models allow capturing (non)linear change over time, comparing differences in shape or degree of change between groups, deal optimally with incomplete data and have improved power to detect associations between change and relevant predictors and covariates by reducing measurement error (Curran et al., 2010, McArdle, 2009). Estimation of individual differences in growth rates, in addition to learning outcomes, can help identify children at risk of developing reading (Yeung, 2018) or math (Xenidou-Dervou et al., 2018) problems. In particular, applying growth models to children's learning data during brief (10–20 min) learning tasks (of decoding skills for example) can serve as an effective method for early assessment of their reading potential. Here growth curve modeling could first be used to relate children’s short-term behavioral learning data to their concurrent neural learning trajectories, to identify (a)typical learning patterns (i.e., individual growth rates McArdle, 2009). Such learning patterns can then be used to predict later change in reading gains, again with growth models. Although applications in the domain of reading are still relatively sparse, latent change score modeling of the number of instructional prompts that children require during a DA of decoding skills at the beginning of first grade, was shown to successfully predict their reading skills at the end of first grade (Cho et al., 2020). If longitudinal data characterizing all stages of skill acquisition (e.g., initial to fluent word decoding skills) are available, multivariate dynamic measurement models (McNeish et al., 2020) can be used to take it one step further and quantify children’s learning potential in terms of expected future achievement scores once the target skill has fully developed. Combined with DA of decoding skills (Cho et al., 2020, Dixon et al., 2023, Gellert and Elbro, 2018), or other explicit (Poulsen and Elbro, 2018, Warmington and Hulme, 2012) and implicit (statistical) learning tasks (Ballan et al., 2023, Vandermosten et al., 2018, Zhang et al., 2021), these models could directly estimate children’s future reading levels and the support required to prevent reading problems. Moreover, children with reading problems show considerable variability in their response to reading intervention (Galuschka et al., 2014). Computational modeling of learning progress and effort across different intervention stages (Christoforou et al., 2023) could be a real game-changer for understanding this variability and, ultimately, for customizing interventions. Indeed it has been argued that a personalized computational model of learning trajectories is essential for a comprehensive explanation of the diverse and individualized nature of dyslexia (Perry et al., 2019). Computational models can provide more fine-grained insights into how learning and decision processes unfold over time on a trial-by-trial basis. This granular perspective facilitates a more precise characterization of diverse learning parameters, leading to a deeper understanding of the underlying mechanisms that drive learning (Christoforou et al., 2023, Fraga-González et al., 2023).

A close examination of various parameters of behavioral and neural learning curves enables pinpointing the phases of learning or the type of process where progress is either hindered or relatively smooth. Such measures may include slope, peak and rate of improvement in accuracy or reaction time trajectories, but also parameters derived from computational models of learning and decision-making, including drift rate, prediction errors, expectations, learning rate, among others (Christoforou et al., 2023, Fraga-González et al., 2023). Combined with empirical models of inter-individual variability, these measures can provide insights into performance disparities across distinct learning phases, including the initial formation of new representations (e.g., letters, letter-sound couplings, phoneme and grapheme clusters, words), their subsequent automatization, or more global differences across learning phases due to variation in attention or working memory abilities. This approach serves as an indicator of a child’s learning potential for specific skills, enabling effective estimation of the type and amount of support a child may require during learning.

While advanced approaches such as, for example, growth modeling have much higher statistical power than comparable traditional methods (Cho et al., 2020, McNeish et al., 2020) and have been successful with samples as small as 22 participants, also here a minimum of 100 participants constitutes a more commonly advised lower bound (Brandmaier et al., 2018, Curran et al., 2010). To obtain models that are both reliable and of practical value in educational settings, our samples not only need to be large and heterogeneous, but also, and probably most importantly, representative of the target group(s). In other words, we need to move beyond our typical biased selection of relatively small groups of children from environments with close affinity to scientific research (Horga et al., 2014). While this already poses a challenge for studies collecting behavioral measures, this is even more so when collecting longitudinal neuroimaging data. Next to finding representative samples, available resources (financial, time, infrastructure) often form a practical bottleneck for the collection of large-scale multifactorial longitudinal data sets. Such large-scale approaches thus require close collaboration across research teams, a prioritization by funding agencies, eventually in coordination with governmental organizations, and, if the project aims for practical applications, a close involvement of stakeholders in education and dyslexia practice from the outset of the project. Such efforts will allow aiming for the collection of much-needed large-scale and multi-level longitudinal data following children’s reading development from kindergarten (or earlier) through primary school years, similar to existing multi-site longitudinal data sets that are being collected in adolescents (e.g., ABCD data set). Ultimately these studies may permit linking genetic and environmental data to multiple levels of neural and behavioral change, across heterogeneous cultural and socioeconomic contexts (Marek et al., 2022, van Atteveldt et al., 2021). Importantly however, especially in newer fields like developmental cognitive neuroscience, exploratory studies that utilize smaller densely phenotyped samples and focus on specific subgroups remain crucial for generating hypotheses. These hypotheses can subsequently be tested using large-scale approaches. Moreover, large consortia projects, due to their scale, tend to focus on general trends and commonalities, potentially overlooking unique or rare cognitive patterns that can be crucial for a comprehensive understanding of individual differences and idiosyncratic trajectories in brain function and cognition.

## Linking short-term to long-term learning trajectories

**Fig. 3:**
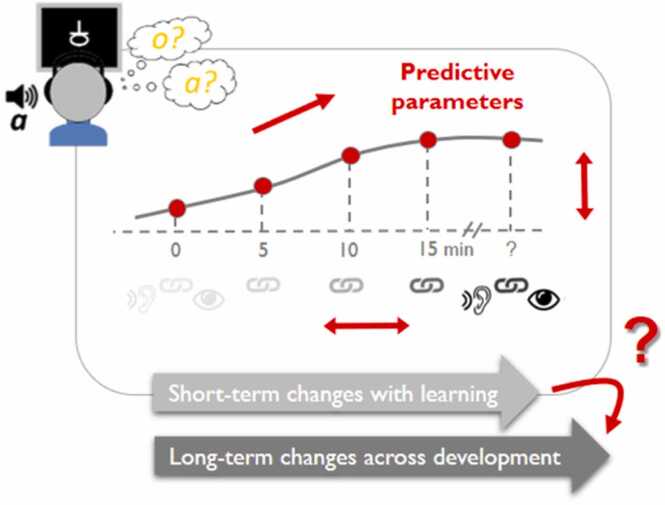
Short-term learning parameters as a proxy of long-term reading development. Schematic illustration of a learning task of early reading skills and corresponding learning parameters (slope, rate, peak) that we hypothesize may predict children’s long-term reading trajectories and outcomes. The given example takes a 15–20 minute learning task of artificial visual symbol - speech sound mappings, akin to one of the core achievements of early reading development: the formation of letter-speech sound mappings (see text for details). Here the use of well-controlled artificial letter-like symbols instead of real letters enables the dynamic assessment of children’s learning potential with minimal bias due to individual differences in prior knowledge/familiarity with letters.

A central hypothesis of our dynamic framework is that the ease at which children learn foundational linguistic operations and representations during short learning tasks forms a proxy for their future reading development. Prime candidates for these learning tasks include decoding skills such as letter - speech sound mapping (see example Fig. 3), blending letters into syllables and words, or visual tuning to letters and letter strings. Other relevant paradigms include paired associate learning (Poulsen and Elbro, 2018, Litt et al., 2013, Warmington and Hulme, 2012), pseudoword or vocabulary learning (Malins et al., 2021, Mayringer and Wimmer, 2000) and implicit statistical learning of linguistic (Vandermosten et al., 2018, Zhang et al., 2021) and non-linguistic (Ballan et al., 2023) information. So far, studies applying learning tasks for outcome prediction still mostly analyzed learning in terms of behavioral outcome scores (Aravena et al., 2018, Gellert and Elbro, 2017, Gellert and Elbro, 2018, Horbach et al., 2015, Horbach et al., 2018) or EEG/fMRI responses ∼2 days after learning (Karipidis et al., 2018, Pleisch et al., 2019b). Employing explicit and implicit learning tasks at different time scales enables quantifying different components of learning. Thus, children’s achieved scores on 10–20 minute learning tasks involving the initial set-up of letter-sound mappings correlate with their current (Aravena et al., 2018, Guerra et al., 2023) and future reading fluency (Gellert and Elbro, 2017, Gellert and Elbro, 2018, Horbach et al., 2015, Horbach et al., 2018, Karipidis et al., 2018). Studying the effects of sleep and longer training enables tapping into consolidation processes. Overnight sleep, for instance, has been shown to boost first graders’ ‘unlearning’ of mirror confusion for letters after a 7.5 hour multisensory-motor training (Torres et al., 2021) and increased children’s new word comprehension the next day after story reading before bedtime (Henderson et al., 2021). Children and adults with reading problems on the other hand, were recently found to show reduced overnight consolidation of newly learned words or pseudowords (Landi et al., 2020; Malins et al., 2021; Smith et al., 2018) and of statistical regularities in tone sequences (Ballan et al., 2023). Such results emphasize that as well as the establishment of specific representations or contents, their consolidation during sleep may also be altered in individuals with reading problems. On a longer time scale, learning tasks can be part of educational DA approaches that integrate cycles of assessment and instruction within reading education and intervention (Grigorenko and Sternberg, 1998, Jeltova et al., 2007).

Next to targeting reading-specific skills, short-term learning tasks can be designed to test the influence of domain-general factors that are central to learning in everyday (school) settings, including e.g., attention (Aravena et al., 2018, Guerra et al., 2023), working memory (Ehm et al., 2019), and socio-emotional factors including self-beliefs and motivation (Verwimp et al., 2023). These tasks may further involve the simulation of various environmental factors that have an impact on learning performance such as noisy classrooms (Guerra et al., 2021) or digital distractions. For optimal future implementation of the learning tasks in educational and/or intervention settings, the tasks can be transformed into digital games, and eventually virtual reality tools, together with game-designers, children and teachers, keeping the motivational and functional needs of both children and teachers at the center of the design process (Scholten and Granic, 2019).

## Summary and conclusions

On the example of reading acquisition and considering the complexity of the neural reading network, here we discuss how various factors, ranging from genes to environmental factors, dynamically shape children’s idiosyncratic learning trajectories, both in terms of behavior and neural processes. We summarize insights from studies at the micro- and macroscopic brain network levels in animals and humans showing that learning involves protracted structural and functional changes at the level of the synapse, neural circuits, and cortical networks. The trajectories that can be quantified based on neurobehavioural measurements during the various stages of learning are often nonlinear and converge with computational accounts of learning, thus highlighting that snapshots or summary measures at a single point in learning, as is commonly done, do often not provide sufficient information for accurate outcome prediction. We emphasize that dense assessments of short-term (learning task) and long-term (development) neural and behavioral measures in large and representative samples across skill learning (e.g., reading) from the novice to the expert stage, are the key to a better understanding and prediction of individual variability. Although we have emphasized the limitations of static performance measures and highlighted the advantages of dynamic approaches for predicting outcomes, we also recognize the enduring high relevance of static measures. Static and dynamic approaches are not mutually exclusive but, rather, complementary. Together with more elaborate statistical analyses techniques such as growth, and normative modeling and dynamic systems approaches such studies may ultimately predict educational outcomes more accurately.

Here we outlined the potential benefits of dynamic, multi-level quantification of learning, particularly in the domain of reading and reading acquisition, and suggest that this dynamic approach also holds promise for applications in various other domains of learning, such as math, language learning, rehabilitation, and clinical interventions (Fuchs et al., 2008). For instance, in multilingual children or patients with aphasia, DA of vocabulary (re)learning can not only help to characterize individual differences in learning potential and progress but also provide valuable insights for clinicians to tailor support (MacLeod and Glaspey, 2022; aphasia Peñaloza et al., 2022; multilingualism Orellana et al., 2019). Likewise also the distal forecast of algebraic or general cognitive abilities benefits from dynamic assessments and in addition will help teachers or therapists to design support for children with specific needs such as those with learning disabilities (Bosma et al., 2017, Fuchs et al., 2011, Fuchs et al., 2008).

In conclusion, while some important initial steps have been taken to use learning as an indicator for long-term developmental outcomes, here we argue that quantifying children's learning-related neurobehavioral changes dynamically during tailored learning tasks will provide a more sensitive and differentiated measure of individual differences in learning capacity. Such an approach can be especially valuable for identifying children who may need additional support and for tailoring interventions to meet their specific needs early on. The development and application of these dynamic, multi-level measures of children’s learning potential rather than their already available knowledge or skills, will critically advance our understanding of how children learn, and, ultimately, will enable teachers in providing optimal and individualized environments for learning and intervention.

## Core concepts

1. Children’s short-term learning trajectories share underlying functional processes with long-term developmental changes.
2. Individual differences in short-term learning trajectories hold crucial information for forecasting future performance.
3. Linking behavioral to neural changes *during* learning tasks of early reading skills will improve quantifying children’s potential for learning to read.

## Research agenda

1. Conduct small- and large-scale longitudinal studies with dense and multi-level data sampling across the time course of learning and development.
2. Design dynamic learning tasks of reading skills and characterize (a)typical learning trajectories through predefined (e.g., developmental dyslexia yes/no) and latent subgroups in the data.
3. Examine which parameters of children’s short-term behavioral and neural learning trajectories are predictive of individual differences in long-term reading trajectories and outcomes.

## Declaration of generative AI and AI-assisted technologies in the writing process

During the preparation of this work the author(s) used ChatGPT3.5 and Bard ai in order to occasionally refine the English phrasing of individual sentences. After using this tool/service, the author(s) reviewed and edited the content as needed and take(s) full responsibility for the content of the publication.

## CRediT authorship contribution statement

**Milene Bonte:** Writing – review & editing, Writing – original draft, Funding acquisition, Conceptualization. **Silvia Brem:** Writing – review & editing, Writing – original draft, Funding acquisition, Conceptualization.

## Declaration of Competing Interest

The authors declare that they have no known competing financial interests or personal relationships that could have appeared to influence the work reported in this paper.
